# Frequency and Characteristics of Overestimated Renal Function in Japanese Patients with Chronic Liver Disease and Its Relation to Sarcopenia

**DOI:** 10.3390/nu13072415

**Published:** 2021-07-14

**Authors:** Sonoe Yoshida, Goki Suda, Masatsugu Ohara, Qingjie Fu, Zijian Yang, Shunichi Hosoda, Megumi Kimura, Kubo Akinori, Yoshimasa Tokuchi, Ren Yamada, Takashi Kitagataya, Kazuharu Suzuki, Naoki Kawagishi, Masato Nakai, Takuya Sho, Mitsuteru Natsuizaka, Kenichi Morikawa, Koji Ogawa, Osamu Maehara, Shunsuke Ohnishi, Naoya Sakamoto

**Affiliations:** 1 Department of Gastroenterology and Hepatology, Graduate School of Medicine, Hokkaido University, Sapporo 060-8638, Japan; sonoeds@pop.med.hokudai.ac.jp (S.Y.); masamasama_zu@yahoo.co.jp (M.O.); fuqingjie@pop.med.hokudai.ac.jp (Q.F.); yuang@eis.hokudai.ac.jp (Z.Y.); hosoda.shunichi.k0@elms.hokudai.ac.jp (S.H.); myamakam@med.hokudai.ac.jp (M.K.); kubo.akinori.q5@elms.hokudai.ac.jp (K.A.); h.y.tokuchi112@med.hokudai.ac.jp (Y.T.); renyama5@pop.med.hokudai.ac.jp (R.Y.); t.kitagataya@pop.med.hokudai.ac.jp (T.K.); kazuharu-s@hospital.hakodate.hokkaido.jp (K.S.); naopaleg@yahoo.co.jp (N.K.); mnakai@pop.med.hokudai.ac.jp (M.N.); shotaku@pop.med.hokudai.ac.jp (T.S.); mitsuteru@natsuizakaclinic.com (M.N.); kenichi.morikawa@med.hokudai.ac.jp (K.M.); k-ogawa@med.hokudai.ac.jp (K.O.); sakamoto@med.hokudai.ac.jp (N.S.); 2 Laboratory of Molecular and Cellular Medicine, Faculty of Pharmaceutical Sciences, Hokkaido University, Sapporo 060-8638, Japan; maeosa17@frontier.hokudai.ac.jp (O.M.); sonishi@pop.med.hokudai.ac.jp (S.O.)

**Keywords:** liver disease, sarcopenia, renal function, creatinine, skeletal muscle

## Abstract

Renal dysfunction and sarcopenia are important prognostic factors in patients with chronic liver disease (CLD). Muscle atrophy can cause the overestimation of renal function based on serum creatinine. However, the frequency of overestimated renal function in Japanese patients with CLD and its relationship with sarcopenia are unclear. In present study, we evaluated the frequency of overestimated renal function, defined as a >20% higher eGFR using creatinine than using cystatin C, in 307 patients with CLD as well as its relationship with indicators of sarcopenia. In total, 24.8% of patients had overestimated renal function. In a multivariate regression analysis, liver cirrhosis (*p* = 0.004) and psoas muscle mass index (*p* = 0.049) were significantly associated with overestimated renal function. Loss of skeletal muscle mass was significantly more frequent in both male and female patients with overestimated renal function than without. In males, the loss of muscle strength and rate of sarcopenia, defined as loss of muscle mass and strength, were significantly higher in patients with than without overestimated renal function. The high frequency of overestimated renal function in Japanese patients suggests that indicators of renal function should be carefully considered; furthermore, monitoring and interventions for both renal function and sarcopenia are needed in patients with CLD.

## 1. Introduction

In patients with chronic liver disease (CLD), renal dysfunction [1,2], and sarcopenia [3,4,5] are important independent prognostic factors; thus, the analysis of the prevalence and characteristics of patients with renal dysfunction or sarcopenia is a clinically important issue.

Primary sarcopenia, defined as age-related skeletal muscle mass atrophy and loss of muscle strength, has a poor prognosis [6,7,8]. CLD is a well-known cause of secondary sarcopenia [8,9]. The Japan Society of Hepatology (JSH) guidelines [10] recommend the diagnosis of sarcopenia in liver disease by measuring muscle strength and skeletal muscle mass. The presence of sarcopenia in patients with CLD is associated with a poor anticancer treatment response [11] and poor prognosis [3].

Serum creatine-based estimated renal function is the most commonly utilized method for the evaluation of renal function. As an important prognostic factor for patients with CLD, serum creatinine levels are included in the model for end-stage liver disease (MELD) scoring system [12]. A loss of skeletal muscle mass frequently results in overestimation of renal function based on estimated glomerular filtration rate (eGFR) using serum creatinine levels in patients with CLD [13]. Because hepatocytes produce creatine, which is mainly stored in muscles, serum creatinine levels could be affected by the loss of skeletal muscle mass in patients with liver disease [13]. However, because all cells produce cystatin C, it is not affected by a loss of muscle mass and liver function [14]. Recent reports have shown that cystatin C-based estimation of renal function is more accurate than creatinine-based assessment in patients with CLD [13]. Quite recently, we found that a loss of skeletal muscle mass causes higher estimates of eGFRcre (eGFR based on creatinine) than eGFRcys (eGFR based on cystatin C) in hepatitis C virus (HCV)-infected patients who received direct-acting antivirals. Yoo et al. evaluated Korean patients with liver cirrhosis (LC) and found that eGFR based on serum creatinine was overestimated in 47% of cases [13]. However, the previous report did not include data for muscle strength, making it difficult to clearly establish the relationship between overestimated renal function and the presence of sarcopenia. Thus, further studies of the overestimation of renal function in Japanese patients with CLD are needed.

In this study, we evaluated the frequency and characteristics of overestimated renal function in patients with CLD and its association with sarcopenia.

## 2. Materials and Methods

### 2.1. Included Patients

Consecutive patients with CLD who visited Hokkaido University Hospital between 2015 and 2018 were screened in the present retrospective study. Patients were included if they were evaluated for muscle mass by the psoas muscle mass index (PMI) using CT, had preserved serum at the time of CT examination, and had proper clinical information. Patients with additional hand grip test data and the presence of sarcopenia were evaluated according to the JSH guidelines [10]. Patients were excluded if they did not undergo a CT examination, did not have preserved serum from the time of the CT examination, underwent hemodialysis, or declined to participate in this study. Patients provided written informed consent to participate and did not decline to participate. 

The following clinical data were collected from patients: sex, age, body mass index (kg/m^2^), laboratory data, Child-Pugh grade, history or stage of hepatocellular carcinoma (HCC), etiology of liver disease, and renal function evaluated by eGFRcre and eGFRcys. LC was diagnosed based on radiologic findings, liver histology, laboratory data, and/or liver stiffness data using FibroScan (Echosens, Paris, France).

The study was conducted according to the guidelines of the Declaration of Helsinki, and approved by the Ethics Committee of Hokkaido University Hospital ethics committee (Clinical Research Number: IRB 016-0397) 

### 2.2. Assessment of Renal Function by Using Serum Creatinine and Cystatin C

Renal function was evaluated using both serum creatinine and cystatin C according to the following equations: eGFRcre (mL/min/1.73 m^2^) = 194 × serum creatinine^−1.094^ × age^−0.287^ × 0.739 (if female) [15] and eGFRcys (mL/min/1. 73 m^2^) = 104 × serum cystatin C − 1.019 × 0.996^age^ (× 0.929 if female) − 8 [16,17]. Overestimation of renal function was defined as a >20% higher value for eGFRcre than eGFRcys, as described previously [13,18,19].

### 2.3. Skeletal Muscle Mass Index Calculation Methods and Definition of Muscle Atrophy

Skeletal muscle mass was calculated using the PMI based on CT images according to previously described methods [18,19,20]. The sum of the L3 level cross-sectional area of the right and left psoas muscle mass was calculated. This value was divided by height squared (cm^2^/m^2^). Muscle atrophy was defined as PMI values of <3.74 cm^2^/m^2^ for men and <2.29 cm^2^/m^2^ for women, as we have described previously [19].

### 2.4. Definition of Loss of Muscle Strength and Sarcopenia

According to the Japan Society of Hepatology (JSH) guidelines for sarcopenia in the liver [10], weak muscle strength was defined as a grip strength of less than 26 kg for men and 18 kg for women. In addition, sarcopenia was defined as a muscle strength decline and loss of muscle mass, similar to the JSH guideline.

### 2.5. Statistical Analysis

Continuous variables were analyzed by Student’s *t*-test or Mann–Whitney U test. Categorical variables were evaluated by Fisher’s exact test. The relationships between two variables were evaluated by Spearman’s rank correlation. Multivariate logistic regression was conducted with variables identified as significant clinical factors (*p* < 0.05) in the univariate analyses. In this study, we utilized Prism 7.03 (GraphPad Software, Inc., La Jolla, CA, USA) for all statistical analyses.

## 3. Results

### 3.1. The Clinical Characteristics That Differed Significantly between Patients with and without Overestimated Renal Function

In total, 307 patients who were analyzed for skeletal muscle mass between January 2015 and December 2018 and had properly preserved serum for analyses of serum cystatin C levels at the time of evaluation of skeletal muscle mass were included (Supplementary Figure S1). The baseline patient characteristics are shown in Table 1. Of the 307 patients, 199 (64.8%) were male and 108 (35.2%) were female. The median age was 68 years (range, 19–90 years), and 82, 64, and 161 patients had CLD of HBV, HCV, and non-B non-C etiologies, respectively. In total, 215 patients (70%) had LC. The overall median PMI values were 3.63 cm^2^/m^2^ (range, 0.71–11.01).

Additional hand grip test data were available in 213 included patients. These patients were evaluated for the presence of sarcopenia (Supplementary Figure S1).

### 3.2. Relationship between eGFRcre, eGFRcys, Serum Creatinine and Cystatin C Levels,

**Figure 1:**
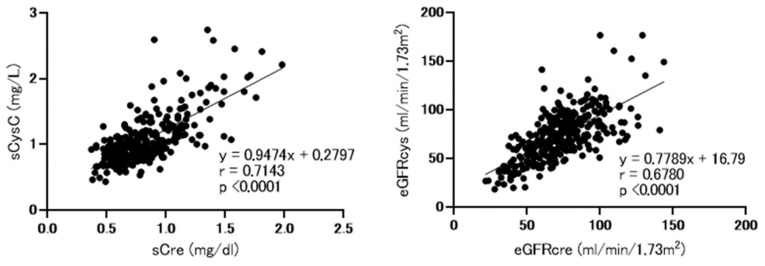
Correlations between baseline serum cystatin C and creatinine levels and between baseline estimated glomerular filtration rate (eGFR) based on cystatin C (eGFRcys) and creatinine (eGFRcre). sCre, serum creatinine; sCysC, serum cystatin C; eGFRcre, estimated glomerular filtration rate based on creatinine, eGFRcys; estimated glomerular filtration rate based on cystatin C. Analysis performed using spearman’s rank correlation test.

As shown in Figure 1, consistent with our previous findings, we detected strong correlations between serum cystatin and creatinine levels and between eGFRcre and eGFRcys [17]. 

### 3.3. Frequency and Characteristics of Patients with Overestimated Renal Function

As shown in Table 1, overestimated renal function based on eGFRcre is observed in 23.8% of patients (76/307). Regarding clinical factors, patients with overestimated renal function had a significantly higher rate of LC (*p* = 0.0019) and lower PMI (*p* = 0.0234) than those of patients without overestimated renal function. Additionally, laboratory data showed that patients with overestimated renal function had significantly lower albumin levels and PT% and higher cystatin C levels (Table 1).

### 3.4. Clinical Factors Associated with Overestimation of Renal Function 

Subsequently, we analyzed clinical factors associated with overestimation of renal function. A multivariate regression analysis performed using variables of clinical factors identified as significant (*p* < 0.05) in the univariate analyses revealed that LC (odds ratio 2.677; 95% confidence interval, 1.359–5.273; *p* = 0.004) and PMI (odds ratio 0.815; 95% confidence interval, 0.665–0.999; *p* = 0.049) were significantly associated with overestimated renal function in patients with CLD (Table 2).

### 3.5. Relationship between Muscle Atrophy and Overestimation of Renal Function

**Figure 2:**
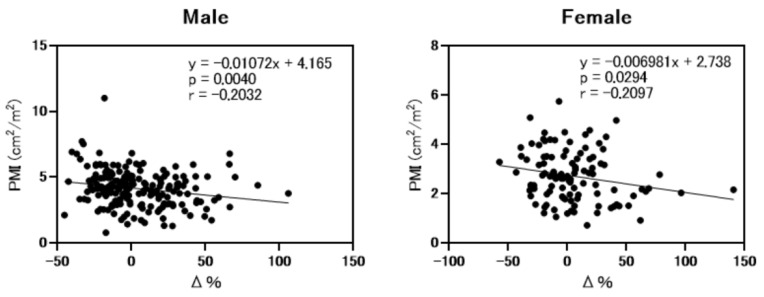
Correlation between psoas muscle mass index and differences between eGFRcre and eGFRcys. PMI; psoas muscle mass index. Δ%; ΔeGFRcre-cys (%) (eGFRcre-eGFRcys/eGFRcre × 100). Analysis performed by Spearman’s rank correlation test.

**Figure 3:**
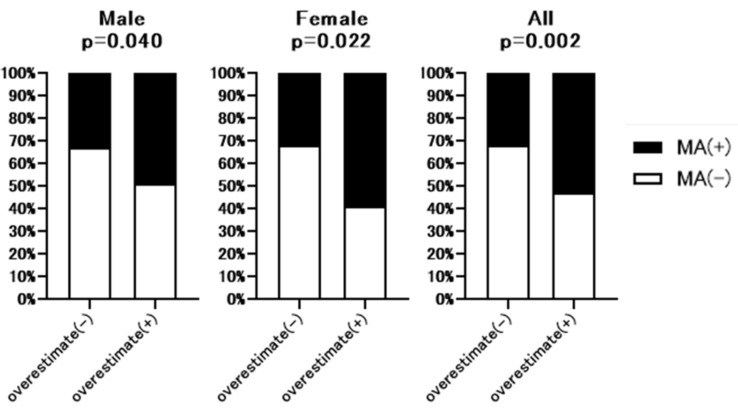
Comparison of the rate of muscle atrophy between patients with or without overestimated renal function. Overestimate (−); patients without overestimated renal function. Overestimate (+); patients with overestimated renal function. MA; muscle atrophy. *p*-value indicates comparison of the Overestimate (+) group vs. Overestimate (−) group.

Next, we analyzed the correlation between ΔeGFRcre-cys (%) (eGFRcre-eGFRcys/eGFRcre × 100) and PMI. As shown in Figure 2, ΔeGFRcre-cys (%) was weakly but significantly correlated with PMI (*r* = −0.2032, *p* = 0.004 in men and *r* = −0.2097, *p* = 0.0294 in female). As shown in Figure 3, the rate of muscle atrophy was significantly higher in patients with overestimated renal function than in those without overestimated renal function for full data set (52.6% vs. 32.5%, *p* = 0.002), males (49.0% vs. 32.7%, *p* = 0.04), and females (59.3% vs. 32.1%, *p* = 0.022). Subsequently, we conducted a subgroup analysis stratified according to advanced liver disease (LC and/or HCC) and age. As shown in Supplementary Table S1, among 307 patients, 250 had advanced liver disease. As shown in Supplementary Figure S2, among these patients, the rate of muscle atrophy was significantly higher in those with renal overestimation (*p* = 0.012). In addition, among the patients without advanced liver disease, the rate of muscle atrophy tended to be higher in patients with overestimated renal dysfunction (*p* = 0.068). As shown in Supplementary Table S2, among 307 patients, 150 were >70 years old, 139 were 50 to 70 years old, and 27 were <50 years old. As shown in Supplementary Figure S3, similar to the overall cohort analysis, in patients >70 years and 50 to 70 years old, the rate of muscle atrophy was significantly higher in those with overestimated renal function (*p* = 0.015 and *p* = 0.016 respectively).

### 3.6. Prevalence of Sarcopenia in Patients with or without Overestimation of Renal Function

**Figure 4:**
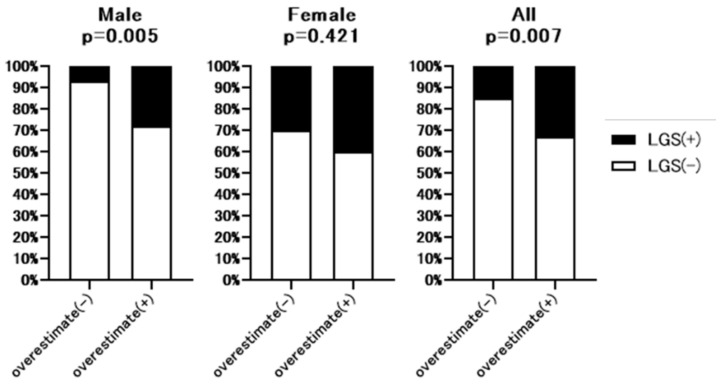
Comparison of the rate of low hand grip strength between patients with or without overestimated renal function. Overestimate (−): patients without overestimated renal function. Overestimate (+): patients with overestimated renal function. LGS: Low hand grip strength. *p*-value indicates comparison of the Overestimate (+) group vs. Overestimate (−) group.

**Figure 5:**
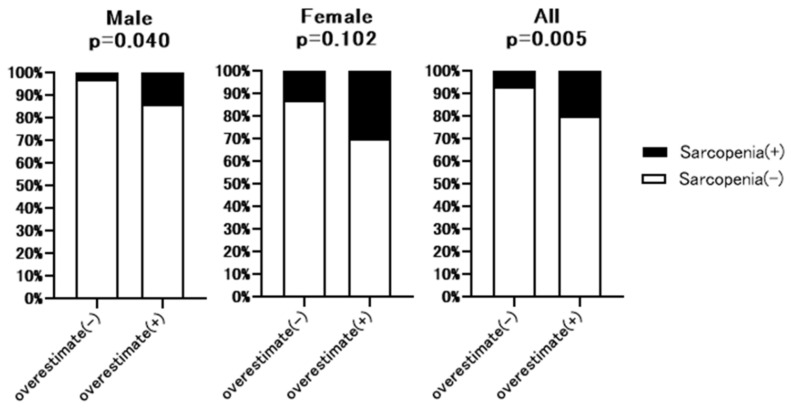
Comparison of the rate of sarcopenia between patients with or without overestimated renal function. Overestimate (−): patients without overestimated renal function. Overestimate (+): patients with overestimated renal function. LGS: Low hand grip strength. *p*-value indicates comparison of the Overestimate (+) group vs. Overestimate (−) group.

Additional hand grip test data were available for 213 of 307 patients. The baseline characteristics of these 213 patents are shown in Supplementary Table S3. As shown in Figure 4, hand grip strength was significantly lower in full dataset (32.7% vs. 15.2%, *p* = 0.007) and male patients (27.6% vs. 6.7%, *p* = 0.005) with overestimated renal function than without overestimated renal function. However, in the female group, there was no significant difference between patients with or without overestimated renal function. Figure 5 summarizes the prevalence of sarcopenia defined by JSH guidelines [10] in patients with overestimated renal function. The rate of sarcopenia was significantly higher in all (20.4% vs. 6.7%, *p* = 0.005) and male (13.8% vs. 2.9%, *p* = 0.04) patients with overestimated renal function than in those without overestimated renal function, with no significant difference in the female group.

## 4. Discussion

In this study, we analyzed the frequency of overestimated renal function in Japanese patients with CLD and the relationship between overestimated renal function, loss of skeletal muscle, and sarcopenia. Overall, 24.8% (76/307) of patients had overestimated renal function. A multivariate analysis of clinical factors revealed that LC and PMI values were significantly associated with overestimated renal function, consistent with the results of a previous study [13]. This result is also consistent with the fact that creatine is produced by hepatocytes and is mainly stored in muscles, resulting in a decrease in serum creatinine levels due to the loss of skeletal muscle mass. The loss of skeletal muscle mass was significantly higher in both females and males with overestimated renal function than without overestimated renal function. Based on additional data for grip hand strength, the loss of muscle strength and rate of sarcopenia were significantly higher in male patients with overestimated renal function than in males without overestimation of renal function. In women, patients with overestimated renal function tended to have a higher rate of sarcopenia than that of patients without overestimated of renal function.

Renal function in patients with CLD, often evaluated by serum creatinine, is an important predictive factor of prognosis [12]. Thus, creatinine is also utilized to calculate the MELD score, which predicts mortality in patients with LC. In our study, prognosis may have been underestimated in 24.8% (76/307) of patients with overestimated renal function based on creatinine. This overestimated renal function was observed regardless of the CKD stage (Supplementary Figure S4). In addition, the dosage of several drugs, including anti-HCV drugs and anti-HBV drugs, needs to be modified depending on renal function; thus, the high frequency of overestimated renal function should be considered to avoid unexpected adverse events in patients with CLD. On the other hand, because all cells produce cystatin C, which is not affected by muscle mass and liver function [14], cystatin C-based evaluation of renal function might be a potential alternative in patients with CLD.

As shown in Figure 3, the rate of muscle atrophy was significantly higher in patients with overestimated renal function. This significant relationship was similarly observed in patients with advanced liver disease and those >70 and 50 to 70 years old, while a similar tendency was observed in patients without advanced liver disease. Conversely, this relationship was not observed in patients <50 years. As the small sample size (*n* = 27) might have affected the results, further analysis is required.

We further established the relationship between overestimated renal function, muscle strength, and sarcopenia in patients with additional handgrip strength data. As shown in Figure 4 and Figure 5, in the male and whole cohorts, the rate of loss of handgrip strength was significantly higher in patients with overestimated renal function than in those without overestimated renal function. Similarly, in men and the whole cohort, the prevalence of sarcopenia was significantly higher in patients with overestimated renal function than in those without overestimated renal function. Although there was a higher rate of sarcopenia in female patients with overestimated renal function, the difference was not significant. It is not clear why the prevalence of sarcopenia and loss of muscle strength differed between females and males with overestimated renal function. One hypothesis is that compared with male patients with CLD, the overall prevalence of loss of muscle strength in female patients was high, making it difficult to detect significant differences between patients with or without overestimated renal function. An alternative hypothesis is that differences in sex hormone levels, including testosterone, which has an anabolic effect, explain the observed differences between males and females [13].

The correlation between PMI and the differences in eGFRcre and eGFRcys were significant, albeit not particularly high. While the precise reason is not clear, there are several hypotheses. As creatine is produced by hepatocytes, the liver’s functional reserve could affect serum creatinine levels, regardless of muscle mass. Such differences in liver functional reserve might affect the results, but further analyses are still required.

Recently, Yoo et al. reported that the frequency of overestimation of eGFR based on serum creatinine exceeds 40% in Korean patients with LC [13]. In the present study, overestimated renal function was detected in 29.8% (64/215) in patients with LC. The older age of patients in this study than in the previous study (54 years vs. 68 years) and the difference in the distribution of etiologies might explain the difference in the prevalence of overestimated renal function in patients with LC. However, the median age of patients in the present study is consistent with that in a recent nationwide analysis of LC in Japan [21]; thus, the data in the present study might accurately reflect Japanese patients with CLD.

In patients with overestimated renal function, the risk of sarcopenia, another poor prognostic factor, is high. Owing to the high frequency of overestimated renal function in patients with CLD, an easy and simple detection method for overestimated renal function might be a useful tool for clinical practice.

The present study had some key strengths. Compared with a previous study [13], we evaluated overestimated renal function, loss of skeletal muscle mass, muscle strength, and sarcopenia simultaneously to clarify the relationships among these factors. However, the present study also had some limitations. Present study was a retrospective single-center study, and the number of included patients, especially patients with data for hand grip strength, was limited. Thus, a prospective study with a larger number of patients is required to validate our results.

In conclusion, our results revealed that a significant proportion of Japanese patients with CLD had overestimated renal function (23.8% (76/307) overall, 29.8% (64/215) in LC, and 13.0% (12/92) in CH). Loss of skeletal muscle mass was significantly associated with overestimation of renal function. Notably, the rate of sarcopenia was higher in male patients with than without overestimated renal function. As renal dysfunction and sarcopenia are independent prognostic factors in patients with CLD, this high rate of overestimated renal function based on creatinine requires careful attention, especially when evaluating the prognosis of these patients. In addition, the dosage of several drugs needs to be modified depending on renal function; thus, the high frequency of overestimated renal function should be considered to avoid unexpected adverse events. On the other hand, cystatin C-based evaluation of renal function might be a potential alternative in patients with CLD.

## Figures and Tables

**Table 1:** Characteristics of patients with or without overestimated renal function.

	Total (*n* = 307)	without Overestimated Renal Function (*n* = 231, 75.2%)	with Overestimated Renal Function (*n* = 76, 24.8%)	*p*-Value
**Clinical Factors**				
Age, years	68 (19–90)	68 (19–88)	70 (43–90)	0.2478
Male/Female	199 (64.8%)/108 (35.2%)	150 (64.9%)/81 (35.1%)	49 (64.5%)/27 (35.5%)	0.9418
CH/LC	92 (30.0%)/215 (70.0%)	80 (34.6%)/151 (65.4%)	12 (15.8%)/64 (84.2%)	0.0019
Etiology, HBV/HCV/NBNC	82 (26.7%)/64 (20.8%)/ 161 (52.4%)	68 (29.4%)/45 (19.5%)/ 118 (51.1%)	14 (18.4%)/19 (25.0%)/ 43 (56.6%)	0.1523
HCC, +/−	142 (46.3%)/165 (53.7%)	108 (46.8%)/123 (53.2%)	34 (44.7%)/42 (55.3%)	0.7597
PMI, cm^2^/m^2^	3.63 (0.71–1.01)	3.75 (0.71–1.01)	3.14 (0.91–6.77)	0.0234
BMI, kg/m^2^	24.3 (14.9–48.9)	24.5 (15.1–48.9)	23.3 (14.9–42.6)	0.2900
**Laboratory Data**				
Platelet count, ×10^4^/mm^3^	13.2 (1.6–66.5)	13.8 (1.6–66.5)	11.4 (2.3–36.8)	0.0910
Prothrombin time, %	84.0 (18.0–148.9)	86.2 (18.0–128.1)	79.3 (32.2–148.9)	0.0332
Serum albumin, g/dL	3.9 (1.8–5.0)	4.0 (1.8–5.0)	3.5 (2.2–4.6)	<0.0001
AST, IU/L	34 (15–210)	32 (15–210)	38 (17–155)	0.0981
ALT, IU/L	25 (8–317)	25 (8–317)	24 (8–214)	0.9459
Creatinine, mg/dL	0.76 (0.37–1.81)	0.79 (0.38–1.98)	0.70 (0.37–1.81)	0.2096
Cystatin C, mg/L	0.96 (0.43–2.74)	0.89 (0.43–2.21)	1.17 (0.81–2.74)	<0.0001

Data are shown as median (range) values or patients’ numbers. Abbreviations: CH, chronic hepatitis; HBV, hepatitis B virus; LC, liver cirrhosis; HCV, hepatitis C virus; NBNC, non-hepatitis B and C virus; PMI, psoas muscle mass index. *p*-value indicates comparison of the without overestimated renal function group vs. with overestimated renal function group.

**Table 2:** Multivariate analysis for clinical factors associated with overestimated renal function in patients with CLD.

	without Overestimated Renal Function	with Overestimated Renal Function	Multivariate Analysis	Odds Ratio 95% (CI)
CH/LC	80/151	12/64	*p* = 0.004	2.677 (1.359–5.273)
PMI (cm^2^/m^2^)	3.75 (0.71–11.01)	3.14 (0.91–6.77)	*p* = 0.049	0.815 (0.665–0.999)

Data are shown as median (range) values. Abbreviations: CH, chronic hepatitis; LC, liver cirrhosis.

## Data Availability

The data that support the findings of this study are available from the corresponding author upon reasonable request.

## Supplementary Materials

The following are available online at https://www.mdpi.com/article/10.3390/nu13072415/s1, Figure S1. Frequency of overestimated renal function depending on CKD stage in patients with chronic liver disease. Overestimation (-); patients without overestimated renal function. Overestimation (+); patients with overestimated renal function. CKD; chronic kidney disease. Table S1. Characteristics of patients with additional data for grip hand strength.
