# Supplementary material for: Frequency and Characteristics of Overestimated Renal Function in Japanese Patients with Chronic Liver Disease and Its Relation to Sarcopenia
Source: Nutrients. 2021 Jul 14;13(7):2415. doi: 10.3390/nu13072415 (PMC8308887; doi:10.3390/nu13072415)
Supplement: Supplementary file 1 [file nutrients-13-02415-s001.zip › nutrients-1249834-supplementary.pdf]

Table S1. Prevalence of overestimated renal function in patients with or without advanced liver disease.

	**Non-advanced Liver Disease (*n* = 57, 18.6%)**	**Advanced Liver Disease (*n* = 250, 81.4%)**	***P*-value**
**Overestimation of Renal Function** +/−	7 (12.3)/50 (87.7)	69 (27.6)/181 (73.4)	0.0156

Table S2. Prevalence of overestimated renal function stratified ages.

	**Age**			***P*-value**
	<50 (*n* = 27, 8.8%)	50–70 (*n* = 139, 45.3%)	70< (*n* = 151, 45.9%)	
**Overestimation of Renal function** +/−	8 (29.6)/ 19 (70.4)	28 (20.1)/111 (79.9)	40 (26.5)/101 (73.5)	0.2323

Table S3. Characteristics of patients with additional data for grip hand strength.

	Total (n = 213)	Without overestimated renal function (n = 164)	With overestimated renal function (n = 49)	P-value
		**Clinical factors**		
**Age, years**	68 (21–90)	68 (21–86)	68 (43–90)	0.6210
**Male / Female**	133 / 80	104 / 60	29 / 20	0.6166
**CH / LC**	57 / 156	48 / 116	9 / 40	0.1451
**Etiology, HBV/HCV/NBNC**	64 / 43 / 106	50 / 35 / 79	14 / 8 / 27	0.6439
**HCC, +/−**	81 / 132	65 / 99	16 / 33	0.4064
**PMI, cm^2/m^2**	3.61 (0.71–6.91)	3.74 (0.71–6.91)	3.03 (0.91–6.77)	0.0234
**Height, cm**	160.8 (136.4–188.7)	161.2 (136.4–188.7)	159.8 (140.4–183.3)	0.3560
**Body weight, kg**	64.0 (32.5–123.0)	65.0 (40.5–123.0)	61.4 (32.5–99.8)	0.1894
**BMI, kg/m^2**	24.4 (14.9–42.6)	24.5 (16.0–39.0)	23.5 (14.9–42.6)	0.4295
**Low Hand Grip Strength, +/−**	41 / 172	25 / 139	16 / 33	0.0120
**Sarcopenia, +/−**	21/ 192	11 / 153	10 / 39	0.0110
		**Laboratory data**		
**Platelet count, $10^4/mm^3$**	13.0 (1.6–66.5)	13.1 (1.6–66.5)	11.9 (2.3–36.8)	0.7023
**Prothrombin time, %**	83.9 (18.0–148.9)	85.1 (18.0–128.1)	79.2 (34.9–148.9)	0.4079
**Serum albumin, g/dL**	4.0 (2.2–5.0)	4.1 (2.4–5.0)	3.6 (2.2–4.6)	<0.0001
**Total Bilirubin, mg/dL**	0.9 (0.3–29.3)	0.8 (0.3–29.3)	1.0 (0.3–18.4)	0.4272
**AST, IU/L**	34 (15–179)	32 (15–179)	37 (17–155)	0.2186
**ALT, IU/L**	25 (8–214)	25 (9–109)	25 (8–214)	0.5109
**Creatinine, mg/dL**	0.75 (0.37–1.76)	0.77 (0.40–1.76)	0.66 (0.37–1.58)	0.0958
**Cystatin C, mg/L**	0.95 (0.49–2.59)	0.90 (0.49–2.05)	1.19 (0.87–2.59)	<0.0001

Data are shown as median (range) values or patients numbers. CPG, Child-Pugh grade, BMI, body mass index; CH, chronic hepatitis; LC, liver cirrhosis; HBV, hepatitis B virus; HCV, hepatitis C virus; NBNC, non-hepatitis B and C virus;HCC, hepatocellular carcinoma; PMI, psoas muscle mass index

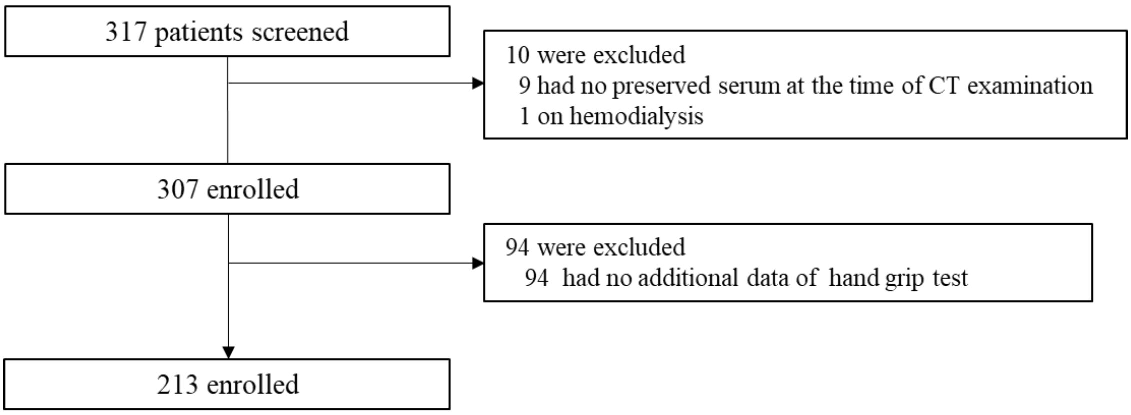

Figure S1. Study Flow.

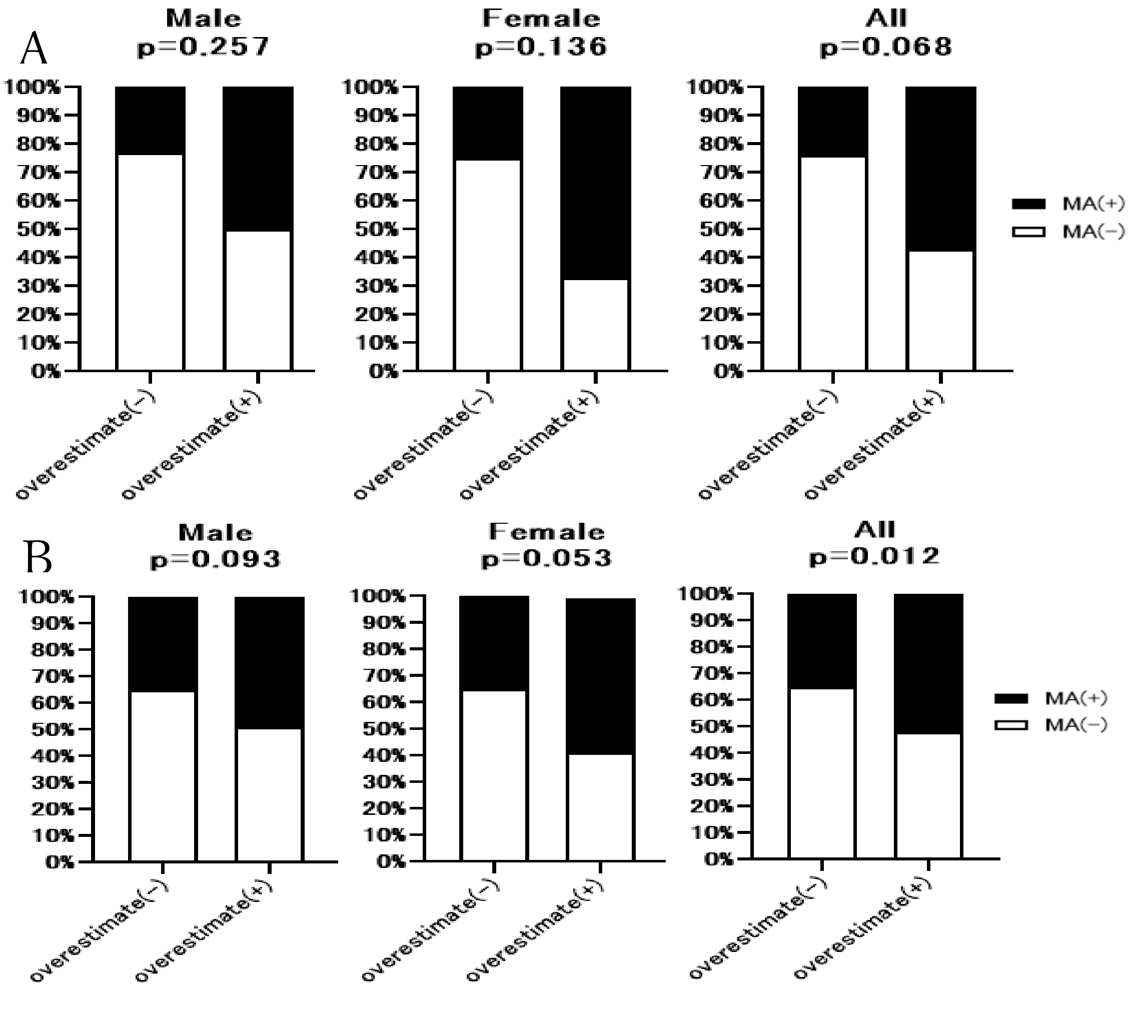

Figure S2. Comparison of the rate of muscle atrophy between patients with or without overestimated renal function, stratified according to advanced liver disease. A. Patients with advanced liver disease. B. Pateitns without advanced liver disease. Overestimate (-), patients without overestimated renal function; Overestimate (+), patients with overestimated renal function; MA, muscle atrophy. P-value indicates comparison of the Overestimate (+) group vs. Overestimate (-) group.

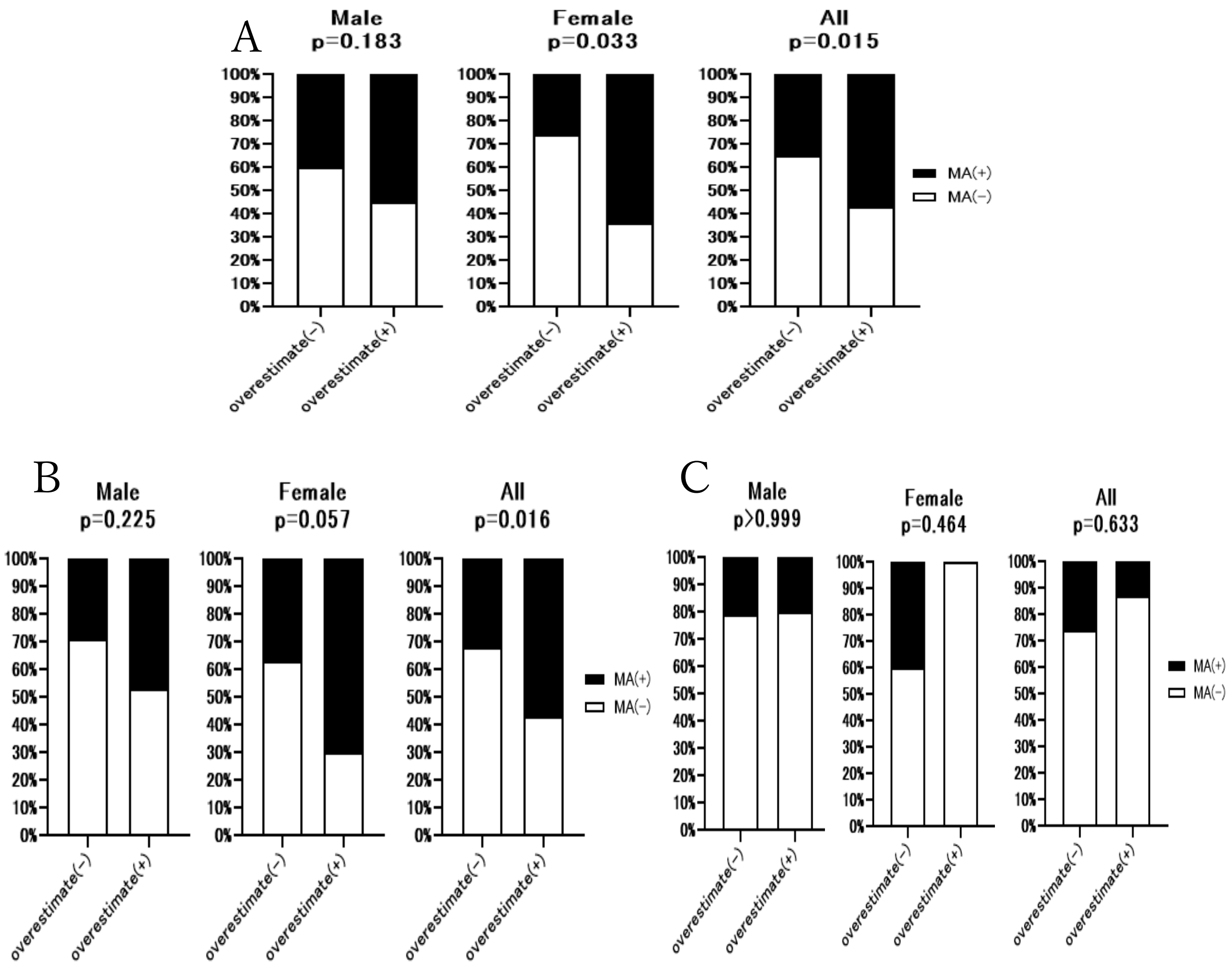

Figure S3. Comparison of the rate of muscle atrophy between patients with or without overestimated renal function, stratified according to age. A. Patients aged >70 years old. B. Pateitns aged 50-70 years old. C. Patients aged <50 years old. Overestimate (-), patients without overestimated renal function; Overestimate (+), patients with overestimated renal function; MA, muscle atrophy. P-value indicates comparison of the Overestimate (+) group vs. Overestimate (-) group.

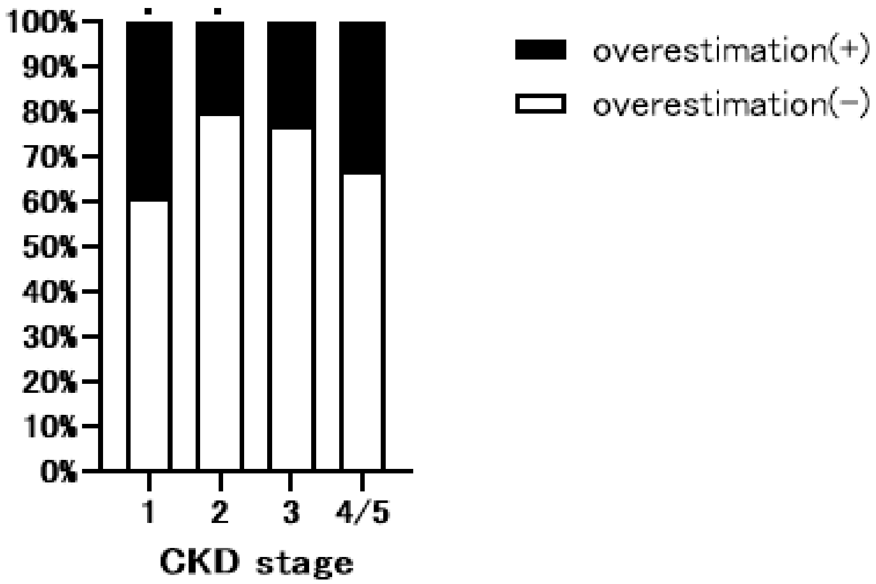

Figure S4. Frequency of overestimated renal function depending on CKD stage in patients with chronic liver disease. Overestimation (-), patients without overestimated renal function**.** Overestimation (+), patients with overestimated renal function. CKD, chronic kidney disease.
